# Energetic Tuning in Spirocyclic Conjugated Polymers

**DOI:** 10.3390/polym8010009

**Published:** 2016-01-06

**Authors:** Hugo Bronstein, Frank D. King

**Affiliations:** Department of Chemistry, University College London, London WC1H 0AJ, UK

**Keywords:** fluorene, spirocyclic, conjugated polymer, xanthene

## Abstract

Precise control of the energy levels in a conjugated polymer is the key to allowing their exploitation in optoelectronic devices. The introduction of spirocycles into conjugated polymers has traditionally been used to enhance their solid state microstructure. Here we present a highly novel method of energetic tuning through the use of electronically active spirocyclic systems. By modifying the size and oxidation state of a heteroatom in an orthogonal spirocycle we demonstrate energetic fine tuning in both the absorption and emission of a conjugated polymer. Furthermore, the synthesis of highly novel triplet-decker spirocyclic conjugated polymers is presented. This new method of energetic manipulation in a conjugated polymer paves the way for future application targeted synthesis of polymers with electronically active spirocycles.

## 1. Introduction

Over the past decades, great efforts have been made to develop new conjugated polymers because of their potential applications particularly in organic light-emitting diodes, solar cells, field effect transistors, charge storage devices, biosensors, and actuators [1]. The key to their success has been their facile electronic and morphological tunability which allows their properties to be systematically varied to suit the needs of the target application. For instance, in organic solar cells the band-gap of the conjugated polymer must simultaneously absorb as much sun-light as possible, whilst retaining sufficient energetic driving force to allow for charge separation at a heterojunction with an electron acceptor [2]. Furthermore, the active layer must have a precise nanostructured morphology so that the resulting optoelectronic processes can occur as rapidly and efficiently as possible [3,4].

One of the most widely used approaches to energetic tunability in conjugated polymers is the donor-acceptor approach whereby orbital hybridization between an electron rich and an electron poor monomer allows for precise tuning of the optical band-gap [5]. Control of thin film nanostructure in conjugated polymers is typically achieved by manipulation of backbone planarity and variation in the attached solubilizing alkyl chains [3,6].

Conjugated polymers with spirocyclic ring systems have been reported multiple times. The use of a tetrahedral center in combination with a planer conjugated backbone has been used to control thin film microstructure and in many cases improve device efficiencies [7,8,9,10]. In particular spirocyclic fluorene and sila-fluorene containing conjugated polymers have displayed enhanced stability and improved emission in comparison to their non-spirocycle containing analogues [9,11]. In organic solar cells, spricocycles are commonly used as electron acceptors [12]. Furthermore, in hybrid solar cells spirocyclic small molecules are the most common form of hole transport materials [13]. Interestingly, small molecule spirocyclic hole transporting materials have also been used to fabricate high efficiency solar cells when present even at low concentrations [14]. However, thus far, the use of spirocycles in conjugated polymers has predominantly been to control their solid state structure, and their potential with regards to the electronic manipulation of the polymer backbone has largely been ignored. In stark contrast, spiro conjugation has been utilized in small molecules to control the frontier molecular orbitals and their respective interactions numerous times [15,16,17,18,19,20].

**Figure 1:**
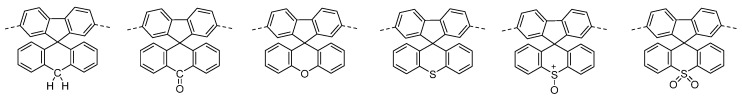
Molecular structure of target spirocycles.

Here we present a series of spirocyclic conjugated polymers, where the orthogonal ring system can be used to tune the electronic structure of the polymer. Thus we envisage a system whereby both electronic and morphological properties can be enhanced simultaneously. Although the polymeric materials presented in this study are more suited towards emissive applications, we believe that this novel method of energetic tuning is applicable throughout all fields of conjugated polymer electronics. To develop this novel strategy we chose to study fluorene containing conjugated polymers due to their ubiquitous use throughout all fields of organic electronics. Furthermore, the use of spirocycle containing fluorene polymers have been extensively studied because of their enhanced chemical stability [9,21]. Of particular interest to us was the report of the spiro-anthracene polymer, which showed high stability and an emission in the deep blue region [22]. We were therefore interested in further investigating similar spiro-systems in which the C-10 atom of the anthracene unit was modified to incorporate both electron donating and electron withdrawing functionality to investigate their effect on the band gap of the polymers. With this in mind we envisaged a series of spiro-based materials that would allow exact elucidation of the role of heteroatom and oxidation state on the properties of a conjugated polymer. The target spirocyclic materials are shown in Figure 1.

In order to maintain solubility, co-polymers with 9,9-dioctylfluorene were targeted. Related to this, a trimer of the spiro-xanthene has been reported to be thermally stable and is a blue-light emitter [23].

## 2. Materials and Methods

9,9-Dioctylfluorene-2,7-diboronic acid bis(1,3-propanediol) ester was obtained from Sigma Aldrich (Poole, UK) and recrystallized from DCM/hexane before use. The other materials were obtained commercially and used as received. Petrol used was petroleum ether of boiling range 40–60 °C.

### 2.1. 10H-Spiro[anthracen-10-one-9,9′-(2′,7′-dibromofluorene)]

To a stirred solution of 10*H*-spiro[anthracene-9,9*′*-(2*′*,7*′*-dibromofluorene)] [22] (3.2 g, 6.6 mmol) in 50 mL of glacial HOAc was added CrO_3_ (2.7 g, 27 mmol) was added at room temperature over 15 min. After stirring at room temperature for 1 h, the reaction mixture was heated to 50 °C for a further 1 h. Ice (50 g) was then added followed by H_2_O (50 mL) and the orange solid collected, washed with H_2_O (3 × 50 mL) and dried. TLC of a solution of the solid showed no starting material (*R*_f_ 0.9, DCM), but a single spot at *R*_f_ 0.5 (DCM). Purification by column chromatography on silica, eluting with 2:1 petrol/DCM gave the title product as a white solid, recrystallized from DCM/petrol (2.5 g, 75% yield). mp: 296–297 °C; ^1^H NMR (600 MHz, CDCl_3_, δ, ppm) 6.54 (2H, d, *J* = 7.8 Hz), 6.98 (2H, d, *J* = 1.7 Hz), 7.34 (2H, dt, *J* = 1.4, 7.6 Hz), 7.45 (1H, dt, *J* = 1.0, 7.5 Hz), 7.53 (2H, dd, *J* = 1.7, 8.2 Hz), 7.71 (2H, d, *J* = 8.2 Hz), 8.45 (2H, dd, *J* = 1.3, 7.9 Hz); ^13^C NMR + DEPT (125Hz, CDCl_3_, δ, ppm) 57.9 (C), 121.9 (CH), 122.8 (C), 127.95 (CH), 127.98 (CH), 128.11 (CH), 128.5 (CH), 131.3 (C), 131.7 (CH), 134.0 (CH), 139.0 (C), 143.0 (C), 154.2 (C), 183.7 (C); FTIR (neat) ν_max_ cm^−1^ 1651, 1599, 1453, 1262, 1249, 813, 687; EI-MS (*m*/*z* %) 505 (MH^+^ 14), 504 (M^+^ 51), 503 (MH^+^ 29), 502 (M^+^ 100), 501 (MH^+^ 16), 500 (M^+^ 50), 313 (40); HRMS (*m*/*z*) Calculated for C_26_H_14_Br_2_O 499.9046 (M^+^), found 499.9043 (M^+^).

### 2.2. 2,7-Dibromospiro(fluorene-9,9′-thioxanthene)

A stirred solution of 1-bromo-2-phenylsulfanylbenzene [24] (1.3 g, 5.0 mmol) in dry Et_2_O (50 mL) under Ar was cooled to −70 °C and a solution of *n*-BuLi (2 mL of 2.5 M in hexanes, 5.0 mmol) was added and the reaction stirred for 30 min. 2,7-Dibromofluorenone (1.3 g, 4.4 mmol) was added and the reaction mixture warmed to room temperature, then stirred for 2 h. Petrol (50 mL) was then added and the solid collected, washed with petrol (2 × 50 mL). The solid was dissolved in THF (10 mL), acidified with 1N HCl (5 mL) and H_2_O (50 mL) added. The solid was collected and dried (1.4 g). The solid was added to glacial HOAc (25 mL), 5 drops of concentrated HCl added and the stirred reaction heated under reflux for 18 h. The reaction mixture was cooled to room temperature and H_2_O (50 mL) added. The solid was collected, washed with H_2_O (2 × 50 mL) and dried. Purification by column chromatography on silica, eluting with petrol, gave the title compound, recrystallized from DCM/petrol as a white solid (0.97 g, 43% yield based upon the dibromo-fluorenone). mp: 229–230 °C; ^1^H NMR (600 MHz, CDCl_3_, δ, ppm) 6.50 (2H, dd, *J* = 1.1, 8.0 Hz), 6.93 (2H, ddd, *J* = 0.06, 1.3, 7.2 Hz), 7.20 (2H, dt, *J* = 1.3, 7.5 Hz), 7.43 (2H, dd, *J* = 1.1, 7.9 Hz), 7.52 (2H, dd, *J* = 1.8, 8.2 Hz), 7.61 (2H, d, *J* = 8.2 Hz), 7.68 (2H, d, *J* = 1.7 Hz); ^13^C NMR + DEPT (125Hz, CDCl_3_, δ, ppm) 60.4 (C), 121.7 (CH), 122.4 (C), 126.5 (CH), 126.6 (CH), 127.8 (CH), 128.2 (CH), 128.9 (CH), 131.0 (C), 131.7 (CH), 135.6 (C), 137.6 (C), 155.8 (C); FTIR (neat) ν_max_ cm^−1^ 1590, 1445, 1408, 1394, 1060, 1044, 874, 801, 744, 722, 696, 651; EI-MS (*m*/*z* %) 509 (MH^+^ 14), 508 (M^+^ 51), 507 (MH^+^ 29), 506 (M^+^ 100), 505 (MH^+^ 16), 504 (M^+^ 52), 425 (35), 345 (37), 173 (26), 172 (25); HRMS (*m*/*z*) Calculated for C_25_H_14_Br_2_S 503.9178 (M^+^), found 503.9179 (M^+^).

### 2.3. 2,7-Dibromospiro(fluorene-9,9′-thioxanthene-S-oxide)

To a stirred solution of 2,7-dibromospiro(fluorene-9,9*′*-thioxanthene) (0.7 g, 1.4 mmol) in CHCl_3_ (25 mL) at −5 °C was added mCPBA (~77%, 0.26 g, ~1.2 mmol) over a period of 10 min and the reaction mixture was stirred for 2 h. Saturated NaHCO_3_ solution (20 mL) was then added. After shaking in a separating funnel, the organic layer was separated, dried (MgSO_4_) and concentrated. TLC of the solution on silica in DCM showed three spots, *R*_f_ 0.9 (starting material), *R*_f_ 0.5 (sulfone) and *R*_f_ 0.1 (sulfoxide). Purification by column chromatography, eluting with DCM gave the title compound, recystallized from CHCl_3_/petrol, as a white solid (0.39 g, 54% yield). mp: 316–317 °C; ^1^H NMR (600 MHz, CDCl_3_, δ, ppm) 6.72 (2H, dd, *J* = 0.9, 8.0 Hz), 7.21 (1H, dd, *J* = 0.3, 1.7 Hz), 7.30 (2H, ddd, *J* = 0.7, 1.4, 7.3 Hz), 7.44 (1H, d, *J* = 1.6 Hz), 7.56 (1H, dd, *J* = 1.7, 8.2 Hz), 7.56 (2H, ddd, *J* = 0.5, 1.1, 7.6 Hz), 7.61 (1H, dd, *J* = 0.3, 8.2 Hz), 7.66 (1H, dd, *J* = 1.8, 8.2 Hz), 7.74 (1H, dd, *J* = 0.3, 8.2 Hz), 8.17 (2H, dd, *J* = 1.1, 7.9 Hz); ^13^C NMR + DEPT (125Hz, CDCl_3_, δ, ppm) 121.9 (CH), 122.1 (CH), 122.3 (C), 122.9 (C), 127.6 (CH), 128.0 (CH), 128.1 (CH), 129.0 (CH), 130.4 (CH), 131.4 (CH), 131.7 (CH), 132.4 (CH), 135.6 (C), 137.5 (C), 139.7 (C), 140.0 (C), 152.3 (C), 153.8 (C); FTIR (neat) ν_max_ cm^−1^ 1454, 1439, 1087, 1062, 1037, 823, 813, 760, 741, 673, 634, 556; EI-MS (*m*/*z* %) 525 (MH^+^ 13), 524 (M^+^ 50), 523 (MH^+^ 30), 522 (M^+^ 93), 521 (MH^+^ 17), 520 (M^+^ 47), 443 (55), 442 (100), 440 (50), 313 (53); HRMS (*m*/*z*) Calculated for C_25_H_14_Br_2_SO 521.9127 (M^+^), found 521.9125 (M^+^).

### 2.4. 2,7-Dibromospiro(fluorene-9,9′-thioxanthene-S,S-dioxide)

A stirred solution of 2,7-dibromospiro(fluorene-9,9*′*-thioxanthene) (0.7 g, 1.4 mmol) and mCPBA (1.1 g, ~4.8 mmol) in CHCl_3_ (25 mL) was heated under reflux for 3 h. TLC (silica, DCM) showed no starting material and a single spot at *R*_f_ 0.5. The reaction mixture was cooled to room temperature and saturated NaHCO_3_ solution (40 mL) was added. After shaking in a separating funnel, the organic layer was separated, dried (MgSO_4_) and concentrated. Purification by column chromatography on silica, eluting with 3:1 DCM/petrol gave the title compound, recrystallized from CHCl_3_/petrol, as a white solid (0.65 g, 87% yield). mp: 247–248 °C; ^1^H NMR (600 MHz, CDCl_3_, δ, ppm) 6.56 (2H, ddd, *J* = 0.7, 1.0, 7.8 Hz), 7.33 (2H, ddd, *J* = 0.9, 1.3, 7.7 Hz), 7.46 (2H, dd, *J* = 0.3, 1.7 Hz), 7.53 (2H, ddd, *J* = 0.9, 1.1, 7.7 Hz), 7.57 (2H, dd, *J* = 1.8, 8.1 Hz), 7.69 (2H, dd, *J* = 0.3, 8.1 Hz), 8.26 (2H, ddd, *J* = 0.3, 1.4, 8.0 Hz); ^13^C NMR + DEPT (125Hz, CDCl_3_, δ, ppm) 57.8 (C), 121.9 (CH), 123.1 (C), 128.9 (CH), 129.0 (CH), 129.2 (CH), 132.3 (CH), 133.2 (CH), 136.8 (C), 138.4 (C), 138.5 (C), 153.9 (C); FTIR (neat) ν_max_ cm^−1^ 1449, 1287, 1159, 1148, 1058, 812, 756, 671, 596, 571; EI-MS (*m*/*z* %) 541 (MH^+^ 14), 540 (M^+^ 56), 539 (MH^+^ 28), 538 (M^+^ 100), 537 (MH^+^ 16), 536 (M^+^ 52), 313 (34), 84 (19); HRMS (*m*/*z*) Calculated for C_25_H_14_Br_2_SO_2_ 535.9076 (M^+^), found 535.9074 (M^+^).

### 2.5. 2,7-Dibromodispiro[fluorene-9,10′-anthracene-9′,9′′-fluorene]

To a stirred solution of 2-bromobiphenyl (0.3 g, 1.3 mmol) in dry Et_2_O (30 mL) under Ar at −10 °C was added a solution of *n*-BuLi (0.5 mL of a 2.5 M solution in hexanes). After 10 min, 10H-Spiro[anthracen-10-one-9,9′-(2′,7′-dibromofluorene)] (0.5 g, 1.0 mmol) was added and the reaction mixture heated under reflux for 30 min. On cooling to room temperature, petrol (50 mL) was added and a few drops of acetone. After stirring for 5 min, 1 M HCl (10 mL) was added and the organic layer separated, dried (MgSO_4_), filtered and concentrate in vacuo to give a white solid that was triturated with petrol, collected, dried and used without further purification. *R*_f_ 0.45 (SiO_2_, 2:1 DCM/petrol); ^1^H NMR (600 MHz, CDCl_3_, δ, ppm) 2.70 (1H, s), 6.10 (2H, d, *J* = 7.3 Hz), 6.22 (2H, d, *J* = 7.9 Hz), 6.34 (1H, s), 6.82 (2H, t, *J* = 7.6 Hz), 6.94–7.00 (3H, m), 7.13–7.18 (4H, m), 7.23 (1H, t, *J* = 7.4 Hz), 7.32 (1H, dd, *J* = 1.7, 8.0 Hz), 7.47 (1H, t, *J* = 7.4 Hz), 7.51 (1H, d, *J* = 8.1 Hz), 7.55 (1H, dd, *J* = 1.7, 8.2 Hz), 7.62 (1H, d, *J* = 8.2 Hz), 7.68 (1H, t, *J* = 7.7 Hz), 7.72 (1H, s), 8.58 (1H, d, *J* = 8.0 Hz); ^13^C NMR + DEPT (125Hz, CDCl_3_, δ, ppm) 59.2 (C), 75.8 (C), 120.7 (CH), 121.5 (CH), 122.2 (C), 122.5 (C), 126.54 (CH), 126.57 (CH), 126.98 (CH), 127.02 (CH), 127.3 (CH), 127.54 (CH), 127.59 (CH), 128.5 (CH), 128.9 (CH), 129.3 (CH), 129.4 (CH), 129.8 (CH), 130.7 (CH), 131.4 (CH), 133.1 (CH), 136.3 (C), 137.4 (C), 140.5 (C), 140.7 (C), 141.78 (C), 141.82 (C), 144.6 (C), 155.5 (C), 156.8 (C). A stirred solution of the carbinol (1.05 g, 1.6 mmol) in HOAc (30 mL) and 4 drops of concentrated HCl was heated under reflux for 1 h. The reaction mixture was cooled, water (100 mL) added and the white solid collected, dried and recystallized from CHCl_3_/MeOH to give the title compound (1.02 g, 100% yield). *R*_f_ 0.3 (SiO_2_, petrol); mp: >350 °C; ^1^H NMR (600 MHz, CDCl_3_, δ, ppm) 6.35–6.39 (2H, m), 6.41–6.44 (2H, m), 6.82–6.86 (4H, m), 7.17 (2H, d, *J* = 7.6 Hz), 7.30–7.34 (4H, m), 7.44 (2H, t, *J* = 7.3 Hz), 7.56 (2H, dd, *J* = 1.7, 8.2 Hz), 7.75 (2H, d, *J* = 8.2 Hz), 7.93 (2H, d, *J* = 7.6 Hz), ^13^C NMR + DEPT (125Hz, CDCl_3_, δ, ppm) 57.8 (C), 58.1 (C), 120.2 (CH), 121.5 (CH), 122.5 (C), 125.3 (CH), 127.1 (CH), 127.4 (CH), 127.7 (CH), 128.6 (CH), 128.7 (CH), 128.9 (CH), 129.1 (CH), 131.1 (CH), 134.7 (C), 136.6 (C), 138.5 (C), 140.5 (C), 156.9 (C), 158.7 (C); FTIR (neat) ν_max_ cm^−1^ 1487, 1444, 813, 753, 731, 672, 641; EI-MS (*m*/*z* %) 641 (MH^+^ 23), 640 (M^+^ 56), 639 (MH^+^ 46), 638 (M^+^ 100), 637 (MH^+^ 30), 636 (M^+^ 50); HRMS (*m*/*z*) Calculated for C_38_H_22_Br_2_ 636.0083 (M^+^), found 636.0081 (M^+^).

### 2.6. Co-polymerisation

The dibromo compounds (0.42 mmol), 9,9-dioctylfluoren-2,7-bis(trimethyleneborate) (0.24 g, 0.42 mmol), Pd_2_(dba)_3_ (9 mg, 0.009 mmol), P(*o*Tol)_3_ (12 mg, 0.036 mmol) and Aliquat 336 (2 drops) were dissolved in toluene (20 mL). The mixture was stirred and thoroughly degassed by alternately evacuating (10 mm Hg) and repleting with argon 4 times, followed by the addition of aqueous Na_2_CO_3_ (5.0 mL of a 1.0 M solution) and evacuating/repleting a further 3 times. The reaction mixture was heated under reflux for 18 h. On cooling, the reaction mixture was poured into MeOH (150 mL) and the solid collected by filtration through celite. After washing with MeOH (2 × 50 mL), the product was dissolved in CHCl_3_ (2 × 100 mL) and filtered. The filtrate was concentrated to ~25 mL, then poured into hexane (150 mL). The solid was collected, washed with hexane (2 × 50 mL) and dried. Yields obtained were in the range of 25%–65%.

## 3. Results

The intermediate di-bromo compounds for polymers **P1** (Vak *et al.*) [22] and **P3** (Xie *et al.*) [23] were prepared by the reported procedures. The ketone for **P2** was prepared from the intermediate for **P1** by CrO_3_ oxidation in a 75% yield. The intermediates for **P4**, **P5** and **P6** was prepared in a manner similar to that of Chan *et al.* in an overall yield of 43% [14]. Low temperature oxidation of this intermediate with 1 equiv. of mCPBA gave the intermediate for **P5** in a 54% yield. Similar oxidation with 2.5 equiv. of mCPBA in CHCl_3_ under reflux gave the intermediate for polymer **P6** in a 87% yield.

**Figure 2:**
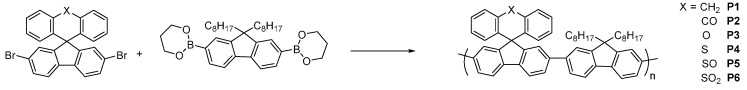
Molecular structure and synthesis of polymers **P1**–**P6**.

The synthetic route of the novel polymers is shown in Figure 2. All novel polymers were synthesized via Suzuki polymerizations using the commercially available di-octyl fluorene boronic ester. Purification of the novel materials was achieved by filtration through a silica plug and repeated precipitations into methanol and hexane. The novel polymers were isolated as green powders and were soluble in common organic solvents such as Toluene and Chloroform.

**Figure 3:**
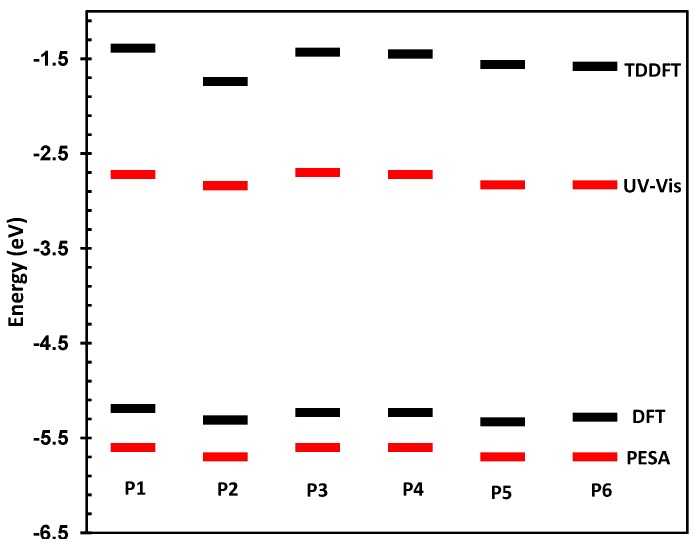
Predicted and measured HOMO and LUMO energy levels for **P1**–**P6**.

The physical and electronic characteristics of the novel polymers are shown in Table 1. All polymers were synthesized with good molecular weights and acceptable PDIs thus allowing for their direct comparison. The HOMO and LUMO of the materials were determined by photoelectron spectroscopy in air (PESA) and their optical band-gaps in thin film. These values are directly compared to those predicted by DFT and TDDFT at the B3LYP/6-31G* level of calculation as has been used for other polymeric systems [25,26]. The comparison between measured and theoretical frontier molecular orbital energy levels is also shown in Figure 3.

**Table 1:** Physical and predicted properties of novel polymers.

Polymer	X	*M_n_* ^a^ /kDa	*M*_w_^a^ /kDa	PDI ^a^	HOMO ^b^ /eV (DFT)	LUMO ^b^ /eV (DFT)	HOMO ^c^ /eV (exp.)	LUMO ^d^ /eV (exp.)	E_gap_^e^ /eV (exp.)
1	CH_2_	10.0	14.2	1.4	−5.19	−1.39	−5.6	−2.7	2.88
2	CO	13.9	24.1	1.7	−5.31	−1.74	−5.7	−2.8	2.86
3	O	12.5	18.9	1.5	−5.23	−1.43	−5.6	−2.7	2.90
4	S	14.9	24.4	1.6	−5.23	−1.45	−5.6	−2.7	2.88
5	SO	15.1	28.9	1.9	−5.33	−1.56	−5.7	−2.8	2.87
6	SO_2_	12.0	20.1	1.7	−5.28	−1.58	−5.7	−2.8	2.87

**^a^** determined by SEC (PS) using PhCl as eluent; **^b^** determined by DFT/TDDFT using B3LYP/6-31G*; **^c^** determined by PESA; **^d^** HOMO + optical energy gap; **^e^** determined from UV–vis.

As can be seen, there is a good correlation between predicted and measured orbital energies, although it must be stated that the error associated with the PESA measurement is typically 0.1 eV. As the variations in the observed HOMO energy levels are relatively small, care must be taken when interpreting results. However, the trends in optical and predicted energy gaps have very good qualitative agreement, thus we can be confident that our chosen level of theory is suitable for discussing the effect of spirocycle on the frontier molecular energy levels. Due to the wide range of substituents present on the synthesized spirocyclic polymers, their effects will be discussed separately in order to derive structure-property relationships and decouple the effects of heteroatom size, electronegativity and oxidation state on the optical properties of the polymers. However, it can clearly be seen that in general, incorporation of an electron deficient spirocycle into a conjugated polymer results in a stabilization of both HOMO and LUMO energy levels. Thus it provides a novel method for controlling important properties such as open circuit voltage in an organic solar cell, and electron and hole injections into OFETs and OLED devices without the need to alter the conjugated polymer backbone.

## 4. Discussion

**Figure 4:**
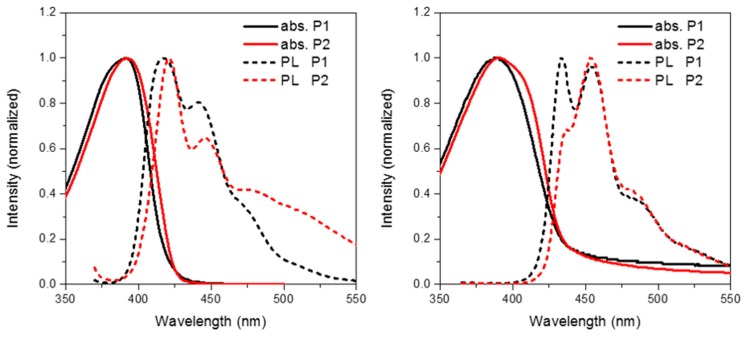
Solution (**left**) and thin film (**right**) absorption and emission spectra of **P1** and **P2**.

**Figure 5:**
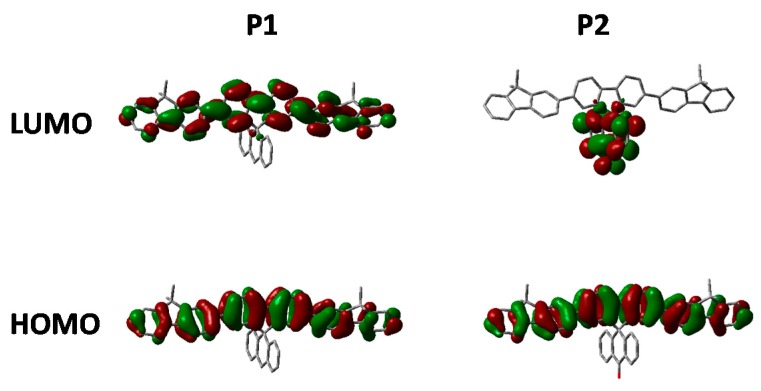
HOMO and LUMO orbitals of **P1** and **P2** (DFT B3LYP/6-31G* isovalue 0.02).

Figure 4 shows the solution (in chloroform) and thin film (spin coated from chlorobenzene) absorption and emission of **P1** and **P2**. The frontier molecule orbitals of an isolated repeat unit (calculated using DFT at the B3LYP/6-31G* level of theory) is shown in Figure 5. From Figure 5 it can clearly be seen that oxidation of the methylene bridge of the spirocycle significantly affects both the HOMO and LUMO of the resulting conjugated polymer. The HOMO is stabilized by approximately 0.1 eV, presumably due to the electron deficient nature of the ketone unit. Similar stabilizations have been observed in conjugated polymers with ketone or ester functionalized side chains [27,28]. As mentioned earlier this may prove to be a novel method to tune the energy of the frontier molecular orbitals in conjugated polymers. Lin *et al.* reported some small molecule spirocyclic materials that contained orthogonal ketone groups which demonstrated good properties as host materials for OLEDs [29]. They reported that a similar stabilization of both HOMO and LUMO was observed relative to a non keto-containing system indicating that the effects present in small molecules are maintained in a conjugated polymer.

The absorption of **P2** relative to **P1** has a similar onset, but it can clearly be seen that there is an additional shoulder present at the longer wavelength in the solid state. Furthermore, in both solution and thin film it can clearly be seen that the absortion of **P2** is redshifted relative to **P1**. We attribute this to the electron deficient nature of its spirocycle resulting in a donor-acceptor type system as is often employed in conventional conjugated polymers for organic solar cell [5]. However, it is interesting to note that similar albeit a smaller effect can also be observed in the spirocyclic system considering the acceptor is orthogonal to the donor.

As can be seen in Figure 5, the electron deficient nature of the spirocycle in **P2** results in a charge-transfer like transition from HOMO to LUMO, as opposed to the more common π–π* type transitions in **P1**. Furthermore, the emission of **P1** and **P2** are significantly different in both solution and solid state. The solution PL of **P1** is reminiscent of polyfluorene which is not surprising considering the electronic neutrality of the attached spirocycle. However, the emission of **P2** is significantly different to that of **P1**. Firstly, the emission onset of **P2** is significantly red-shifted relative to **P1** in both solution and thin film. In solution, the PL of **P2** displays a new red-shifted broad emissive feature which we tentatively assign to emission from a charge-transfer like state. It is interesting to note that the separation between ground and excited state observed in **P2** is not dissimilar to that observed in small molecules for thermally activated delayed fluorescence and could prove an important tool for excited state manipulation in other conjugated materials [30,31]. However, it must be noted that this emissive feature is not too dissimilar from the “keto-defect” emission that is sometimes observed in polyfluorenes [32]. We believe it is unlikely that the origin of this emission is unlikely to originate from keto-defects in the fluorine co-monomer as the same batch was used for the synthesis of all the polymers, none others of which display this broad emission. However, the presence of the ketone group on the spirocycle may be inducing excimer formation which is thought to be the origin of the green emission in keto defect PFO [33]. In the solid state, emission of **P1** is again similar to that of polyfluorene, however **P2** again displays significantly altered features. The broad emission is no longer present suggesting that the emission does not originate from keto defects, but instead suggests that the local environment is not sufficiently polar to stabilize the proposed C-T state. Similar changes in C-T emissions have been seen in small molecules with orthogonal donor-acceptor systems, but have rarely been reported in conjugated polymers [34,35]. The emission profile displays a significantly smaller contribution from the 0–0 transition again potentially due to some kind of charge-transfer like excited state which results in significant geometrical changes in the excited state resulting in reduced frank-condon overlap during emission.

**Figure 6:**
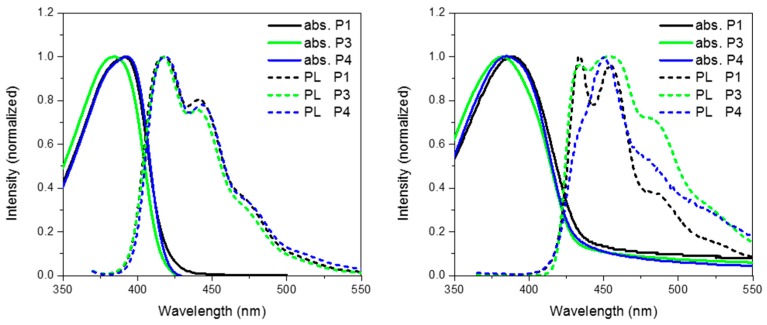
Thin film absorption, emission and HOMO and LUMO of **P3** and **P4**.

Figure 6 show the absorption and emission of **P3** and **P4** solution (CHCl_3_) and thin film (spin coated from chlorobenzene). As can be seen from Table 1 and the absorption spectra, replacement of CH_2_ for either O or S has no major effect on the ground state properties of the spirocyclic polymers. However, it can clearly be seen that the xanthene substituted conjugated polymer (**P3**) has a blue-shifted absorption maxima indicating a wider band-gap. The frontier molecular orbitals are shown in Figure 6. It can be seen that there is some LUMO density on the orthogonal pi system, whereas the HOMO is completely localized on the polymer backbone. Therefore, it is suggested that the oxygen atom of the spiro-xanthene can act as a weak donor resulting in destabilization of the LUMO and hence a wider band-gap. The distorted nature of the thio-xanthene polymer (**P4**) may prevent effective orbital overlap. In solution, the emission of **P1**, **P3** and **P4** are virtually identical indicating that they have similar excited state properties. However, the photoluminescent spectra of the polymers in the solid state differ significantly indicating that heteroatom substitution in fact greatly affects the solid state packing of the polymers. The emission of **P3** as compared to **P1** is significantly broader, but retains almost identical features. Spiro-xanthene containing small molecules have been reported previously in the literature for a variety of uses in the field of organic electronics, but typically their emission spectra retain sharp spectra features [36,37,38]. However, in one example of a hole transporting spiro-xanthene small molecule and in a highly crosslinked amorphous spiroxanthene containing system, broad featureless emission has been observed [7,39]. **P4** displays significantly weaker emission from the lowest vibrational excited state when compared to both **P1** and **P3**.

**Figure 7:**
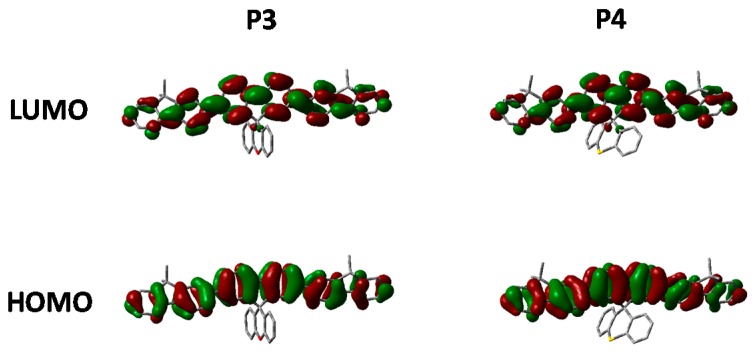
HOMO and LUMO orbitals of **P3** and **P4** (DFT B3LYP/6-31G* isovalue 0.02).

Comparing the optimized geometry and frontier molecular orbitals in Figure 7 significant differences can be observed. The nature of the electronic transitions appear to be the same for all three polymers with minimal contribution from the spirocycle. However, the optimized geometry for **P4** is significantly distorted when compared to **P1** and **P3**. We suggest that the larger size of the S atom in **P4** prevents the normally tetrahedral arrangement of the spirocycle from being the lowest stable conformation. Similar structural deviations have also been reported for small molecule thio-xanthene and related materials [40]. We suggest that after initial photoexcitation this strained geometry results in greater relaxation than in **P1** and **P3**, and hence a greater deviation from the ground state geometry with the result that emission from the lowest vibrational state is no longer favorable. This series of materials therefore demonstrates a highly novel method to manipulate excited state properties, whilst maintaining a similar ground state and hence may also prove a useful tool for controlling excitons in conjugated polymers.

**Figure 8:**
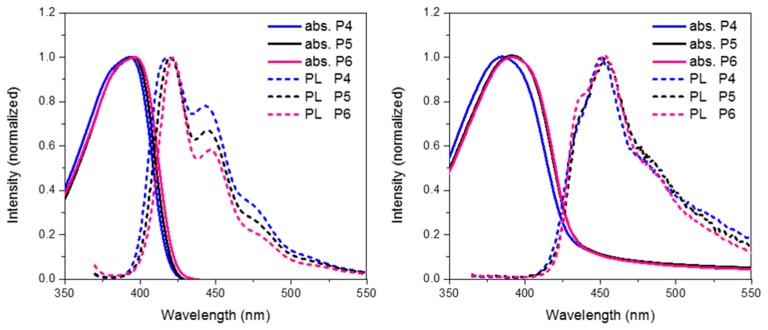
Thin film absorption, emission and HOMO and LUMO of **P5** and **P6**.

Finally we compare the effect of oxidation state on the optoelectronic properties of spirocyclic polymer systems. Figure 8 show the absorption, emission for **P4**, **P5** and **P6** in solution and solid state. In general, all 3 polymers display similar optical properties. In solution it can be seen that there is a slight red-shift on increasing the electron withdrawing nature of the orthogonal spirocycle. In thin film the optical energy gaps for **P5** and **P6** are both very similar displaying a clear red-shift in the absorption maxima relative to the parent polymer **P4**. The observed red-shift in absorption is likely to be due to the more electron deficient nature of their respective spirocycles leading to some degree of “donor-acceptor” type interactions. All 3 polymers display similar emissive behavior in both solution and thin-film with the only significant difference being that in solution there are smaller contributions from lower energy features going from **P4** to **P6**.

**Figure 9:**
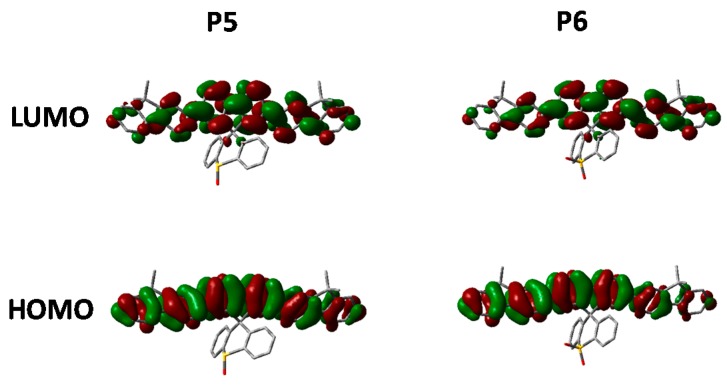
HOMO and LUMO orbitals of **P5** and **P6** (DFT B3LYP/6-31G* isovalue 0.02).

Looking at the optimized geometries (Figure 9) it can clearly be seen that all three polymers display the same degree of distortion from the ideal tetrahedral spirocyclic ring system. As mentioned previously this is likely to be due to the larger size of S relative to C or O, and has also been reported in the literature [16,18,40]. However, the increased electron withdrawing nature of the SO and SO_2_ groups results in an energetic stabilization of both the HOMO and LUMO of approximately 0.1 eV. Furthermore, the emissive properties of all three polymers is very similar indicating that the effect of oxidation state on the frontier molecular orbitals is primarily inductive as opposed to those observed in **P2**. The absence of any kind of charge-transfer like transition in **P5** and **P6** is interesting considering that in small molecule containing oxo and dioxo thioxanthene containing spirocycles, their electron deficient nature often results in very strong C-T type interactions [17,18]. We suggest that the absence of this type of transition is due to the relatively weak donating effect of the peripheral fluorine units, as opposed to the typically more electron rich groups reported in the small molecule literature. Therefore, it appears that in conjugated polymers orthogonal spirocyclic oxidized xanthenes can be used to inductively tune the band-gap whilst not being directly involved in the optical transitions.

Overall, it can clearly be seen that both the size and electronegativity of orthogonal spirocyclic systems can be used to tune both the ground and excited state of a conjugated polymer, giving the synthetic chemist a novel method to engineer new materials for optoelectronic applications.

**Figure 10:**
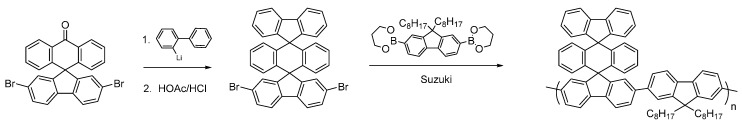
Synthesis of novel triple-decker spirocyclic conjugated polymer.

Having synthesized the dibromoketone for **P4**, we decide to investigate polymers containing a highly novel bis-spirocyclic system. Triple-decker spirocyclic systems have been reported previously but to the best of our knowledge they have never been incorporated into conjugated polymers [41]. The intermediate dibromo-compound was synthesized via addition of 2-lithiobiphenyl to 2,7-dibromofluoerenone to give the carbinol, which was cyclized with concentrated HCl in acetic acid in an overall yield of 83% (Figure 10) in a similar fashion to that reported by Cho *et al.* [41]. Suzuki polymerization with dioctylfluorene afforded the novel triple-decker polymer with a molecular weight of *M*_n_ ~ 17 kDa and a PDI of 1.7. This novel triple-decker bis-spiro conjugated polymer will form the basis for future investigations to incorporate electron-withdrawing substituents.

## 5. Conclusions

We have demonstrated novel synthetic methodology towards spirocyclic conjugated polymers for use in optoelectronic applications. Heteroatomic and oxidative variations of the spirocycle allow for subtle tuning of the ground and excited states to be achieved. Specifically, both inductive and conformational effects can be exploited to tune both the HOMO and LUMO of conjugated polymers. Furthermore, both the absorption and the emission of the novel materials can be tuned in-dependently. Finally, we present the synthesis of previously unreported bis-spirocyclic conjugated polymers. We believe that these developments will allow for the precise tuning of electroactive polymers allowing for further progress to be made throughout the field of organic electronics.

## References

[1] Facchetti A. (2011). Π-conjugated polymers for organic electronics and photovoltaic cell applications. Chem. Mater..

[2] Bronstein H., Collado-Fregoso E., Hadipour A., Soon Y.W., Huang Z., Dimitrov S.D., Ashraf R.S., Rand B.P., Watkins S.E., Tuladhar P.S. (2013). Thieno[3,2-b]thiophene-diketopyrrolopyrrole containing polymers for inverted solar cells devices with high short circuit currents. Adv. Funct. Mater..

[3] Bronstein H., Leem D.S., Hamilton R., Woebkenberg P., King S., Zhang W., Ashraf R.S., Heeney M., Anthopoulos T.D., Mello J.D. (2011). Indacenodithiophene-*co*-benzothiadiazole copolymers for high performance solar cells or transistors via alkyl chain optimization. Macromolecules.

[4] Brabec C.J., Heeney M., McCulloch I., Nelson J. (2011). Influence of blend microstructure on bulk heterojunction organic photovoltaic performance. Chem. Soc. Rev..

[5] Zhang Z.-G., Wang J. (2012). Structures and properties of conjugated donor-acceptor copolymers for solar cell applications. J. Mater. Chem..

[6] Meager I., Ashraf R.S., Rossbauer S., Bronstein H., Donaghey J.E., Marshall J., Schroeder B.C., Heeney M., Anthopoulos T.D., McCulloch I. (2013). Alkyl chain extension as a route to novel thieno[3,2-*b*]thiophene flanked diketopyrrolopyrrole polymers for use in organic solar cells and field effect transistors. Macromolecules.

[7] Chen Q., Wang J.-X., Wang Q., Bian N., Li Z.-H., Yan C.-G., Han B.-H. (2011). Spiro(fluorene-9,9′-xanthene)-based porous organic polymers: Preparation, porosity, and exceptional hydrogen uptake at low pressure. Macromolecules.

[8] Wu F.-I., Dodda R., Reddy D.S., Shu C.-F. (2002). Synthesis and characterization of spiro-linked poly(terfluorene): A blue-emitting polymer with controlled conjugated length. J. Mater. Chem..

[9] Yu W.L., Pei J., Huang W., Heeger A.J. (2000). Spiro-functionalized polyfluorene derivatives as blue light-emitting materials. Adv. Mater..

[10] McFarlane S.L., Coumont L.S., Piercey D.G., McDonald R., Veinot J.G.C. (2008). “One-pot” synthesis of a thermally stable blue emitter: Poly[spiro(fluorene-9,9′-(2′-phenoxy-xanthene)]. Macromolecules.

[11] McDowell J.J., Gao D., Seferos D.S., Ozin G. (2015). Synthesis of poly(spirosilabifluorene) copolymers and their improved stability in blue emitting polymer leds over non-spiro analogs. Polym. Chem..

[12] Xia D., Gehrig D., Guo X., Baumgarten M., Laquai F., Mullen K. (2015). A spiro-bifluorene based 3D electron acceptor with dicyanovinylene substitution for solution-processed non-fullerene organic solar cells. J. Mater. Chem. A.

[13] Jeon N.J., Lee H.G., Kim Y.C., Seo J., Noh J.H., Lee J., Seok S.I. (2014). *O*-methoxy substituents in spiro-ometad for efficient inorganic–organic hybrid perovskite solar cells. J. Am. Chem. Soc..

[14] Chan C.-Y., Wong Y.-C., Chan M.-Y., Cheung S.-H., So S.-K., Yam V.W.-W. (2014). Hole-transporting spirothioxanthene derivatives as donor materials for efficient small-molecule-based organic photovoltaic devices. Chem. Mater..

[15] Maslak P. (1994). Spiroconjugation: An added dimensi in the design of organic molecular materials. Adv. Mater..

[16] Li Y., Wang Z., Li X., Xie G., Chen D., Wang Y.-F., Lo C.-C., Lien A., Peng J., Cao Y. (2015). Highly efficient spiro[fluorene-9,9′-thioxanthene] core derived blue emitters and fluorescent/phosphorescent hybrid white organic light-emitting diodes. Chem. Mater..

[17] Romain M., Tondelier D., Geffroy B., Shirinskaya A., Jeannin O., Rault-Berthelot J., Poriel C. (2015). Spiro-configured phenyl acridine thioxanthene dioxide as a host for efficient pholeds. Chem. Commun..

[18] Romain M., Tondelier D., Jeannin O., Geffroy B., Rault-Berthelot J., Poriel C. (2015). Properties modulation of organic semi-conductors based on a donor-spiro-acceptor (D-spiro-A) molecular design: New host materials for efficient sky-blue PhOLEDs. J. Mater. Chem. C.

[19] Yao C., Yu Y., Yang X., Zhang H., Huang Z., Xu X., Zhou G., Yue L., Wu Z. (2015). Effective blocking of the molecular aggregation of novel truxene-based emitters with spirobifluorene and electron-donating moieties for furnishing highly efficient non-doped blue-emitting oleds. J. Mater. Chem. C.

[20] Shao S., Ma Z., Ding J., Wang L., Jing X., Wang F. (2012). Spiro-linked hyperbranched architecture in electrophosphorescent conjugated polymers for tailoring triplet energy back transfer. Adv. Mater..

[21] Wang H.-Y., Qian Q., Lin K.-H., Peng B., Huang W., Liu F., Wei W. (2012). Stable and good color purity white light-emitting devices based on random fluorene/spirofluorene copolymers doped with iridium complex. J. Polym. Sci. Polym. Phys..

[22] Vak D., Chun C., Lee C.L., Kim J.-J., Kim D.-Y. (2004). A novel spiro-functionalized polyfluorene derivative with solubilizing side chains. J. Mater. Chem..

[23] Xie L.-H., Liu F., Tang C., Hou X.-Y., Hua Y.-R., Fan Q.-L., Huang W. (2006). Unexpected one-pot method to synthesize spiro[fluorene-9,9′-xanthene] building blocks for blue-light-emitting materials. Org. Lett..

[24] Capozzi M.A.M., Centrone C., Fracchiolla G., Naso F., Cardellicchio C. (2011). A study of factors affecting enantioselectivity in the oxidation of aryl benzyl sulfides in the presence of chiral titanium catalysts. Eur. J. Org. Chem..

[25] Bronstein H., Ashraf R.S., Kim Y., White A.J.P., Anthopoulos T., Song K., James D., Zhang W., McCulloch I. (2011). Synthesis of a novel fused thiophene-thieno 3,2-*b* thiophene-thiophene donor monomer and co-polymer for use in opv and ofets. Macromol. Rapid Commun..

[26] Bronstein H.A., Finlayson C.E., Kirov K.R., Friend R.H., Williams C.K. (2008). Investigation into the phosphorescence of a series of regioisomeric iridium(III) complexes. Organometallics.

[27] Chen L., Shen X., Chen Y. (2012). A novel thiophene derivative-based conjugated polymer for polymer solar cells with high open-circuit voltage. Chin. J. Chem..

[28] Bridges C.R., Guo C., Yan H., Miltenburg M.B., Li P., Li Y., Seferos D.S. (2015). Conjugated polymers with switchable carrier polarity. Macromolecules.

[29] Chi-Jen L., Heh-Lung H., Mei-Rurng T., Cheng C.-H. (2009). High energy gap OLED host materials for green and blue PhOLED materials. J. Disp. Technol..

[30] Nakagawa T., Ku S.-Y., Wong K.-T., Adachi C. (2012). Electroluminescence based on thermally activated delayed fluorescence generated by a spirobifluorene donor-acceptor structure. Chem. Commun..

[31] Zhang Q., Li B., Huang S., Nomura H., Tanaka H., Adachi C. (2014). Efficient blue organic light-emitting diodes employing thermally activated delayed fluorescence. Nat. Photon..

[32] Noh Y.-Y., Kim D.-Y., Yoshida Y., Yase K., Jung B.-J., Lim E., Shim H.-K., Azumi R. (2005). Keto defect sites in fluorene-based organic field-effect transistors: The origin of rapid degradation on the performance of the device. J. Appl. Phys..

[33] Lupton J.M., Craig M.R., Meijer E.W. (2002). On-chain defect emission in electroluminescent polyfluorenes. Appl. Phys. Lett..

[34] Jankus V., Data P., Graves D., McGuinness C., Santos J., Bryce M.R., Dias F.B., Monkman A.P. (2014). Highly efficient TADF OLEDs: How the emitter–host interaction controls both the excited state species and electrical properties of the devices to achieve near 100% triplet harvesting and high efficiency. Adv. Funct. Mater..

[35] Dias F.B., Pollock S., Hedley G., Pålsson L.-O., Monkman A., Perepichka I.I., Perepichka I.F., Tavasli M., Bryce M.R. (2006). Intramolecular charge transfer assisted by conformational changes in the excited state of fluorene-dibenzothiophene-*S*,*S*-dioxide co-oligomers. J. Phys. Chem. B.

[36] Poriel C., Cocherel N., Rault-Berthelot J., Vignau L., Jeannin O. (2011). Incorporation of spiroxanthene units in blue-emitting oligophenylene frameworks: A new molecular design for OLED applications. Chem. Eur. J..

[37] Cocherel N., Poriel C., Vignau L., Bergamini J.-F., Rault-Berthelot J. (2010). Dispiroxanthene-indenofluorene: A new blue emitter for nondoped organic light emitting diode applications. Org. Lett..

[38] Chu Z., Wang D., Zhang C., Wang F., Wu H., Lv Z., Hou S., Fan X., Zou D. (2012). Synthesis of spiro[fluorene-9,9′-xanthene] derivatives and their application as hole-transporting materials for organic light-emitting devices. Synth. Met..

[39] Gu J.-F., Xie G.-H., Zhang L., Chen S.-F., Lin Z.-Q., Zhang Z.-S., Zhao J.-F., Xie L.-H., Tang C., Zhao Y. (2010). Dumbbell-shaped spirocyclic aromatic hydrocarbon to control intermolecular π−π stacking interaction for high-performance nondoped deep-blue organic light-emitting devices. J. Phys. Chem. Lett..

[40] Yao L., Sun S., Xue S., Zhang S., Wu X., Zhang H., Pan Y., Gu C., Li F., Ma Y. (2013). Aromatic s-heterocycle and fluorene derivatives as solution-processed blue fluorescent emitters: Structure–property relationships for different sulfur oxidation states. J. Phys. Chem. C.

[41] Cho Y.J., Kim O.Y., Lee J.Y. (2012). Synthesis of an aromatic amine derivative with novel double spirobifluorene core and its application as a hole transport material. Org. Electron..

